# Can vitamin B6 alleviate the adverse reactions of quadruple anti-*Helicobacter pylori* regimen? : randomized controlled trial

**DOI:** 10.1186/s12879-023-08571-8

**Published:** 2023-09-11

**Authors:** Meng-Yan Cui, Meng-Jie Zhang, Qiao-Li Jiang, Zhong-Mei Pei, Zhen-Yu Cui, Mei Kang, Lun-Gen Lu, Ying-Ying Lu

**Affiliations:** 1 Shanghai Key Laboratory of Pancreatic Disease, Shanghai Jiao Tong University School of Medicine, Shanghai, 201620 China; 2 Department of Gastroenterology, Shanghai General Hospital, Shanghai Jiao Tong University School of Medicine, Shanghai, 201620 China; 3 Department of Gastroenterology, Anhui Provincial Key Laboratory of Digestive Disease, The First Affiliated Hospital of Anhui Medical University, Hefei, 230032 China; 4 Department of Gastroenterology, Jiading branch of Shanghai General Hospital, Shanghai Jiao Tong University School of Medicine, 800 Huangjiahuayuan Road, Shanghai, 201803 China; 5 Clinical Research Center, Shanghai General Hospital, Shanghai Jiao Tong University School of Medicine, Shanghai, 200080 China

**Keywords:** Anti-*Helicobacter pylori* regimen, Vitamin B6, Adverse reactions, Minocycline, Metronidazole

## Abstract

**Background:**

Vitamin B6 is an essential water-soluble vitamin for humans. It is often used to prevent a variety of neuropathies, relieve vomiting, and relieve symptoms such as hand and foot neuritis.

**Aim:**

To evaluate whether vitamin B6 can alleviate the adverse reactions caused by the quadruple anti-*Helicobacter pylori* treatment regimen containing minocycline and metronidazole.

**Methods:**

In this randomized controlled trial, 280 patients with *H. pylori* infection were randomly placed into one of two treatment groups—the conventional treatment group and the vitamin B6 supplement treatment group—for 2 weeks. The primary endpoint was the total incidence of adverse reactions up to 2 weeks after treatment initiation. The study was designed according to CONSORT Medicinal Interventions. And it was registered with Chinese Clinical Trial Registry under the number ChiCTR2100053833.

**Results:**

In terms of efficacy, vitamin B6 does not affect the efficacy of conventional regimen. In the vitamin B6 supplement treatment group, the incidence of adverse reactions was 56.92%, which was significantly lower than the 74.62% observed in the conventional treatment group. In addition, the severity of adverse reactions was also significantly reduced. The proportion of moderate to severe central nervous system symptoms decreased from 58.7 to 14.63%. And, the proportion of moderate to severe gastrointestinal reactions decreased from 33.33 to 0%. We speculate that the mechanism of vitamin B6 of reducing adverse reaction may be related to the production of GABA in the brain.

**Conclusions:**

Vitamin B6 can alleviate adverse reactions of the quadruple anti-*H. pylori* regimen containing minocycline and metronidazole.

**Supplementary Information:**

The online version contains supplementary material available at 10.1186/s12879-023-08571-8.

## Introduction

*Helicobacter pylori* (*H. pylori*) infection is one of the most common bacterial infections, affecting approximately 50% of the global population [1]. For people with *H. pylori* infection, eradication therapy can relieve clinical symptoms, improve the quality of life, and reduce cancer risk [2]. Therefore, eradication of *H. pylori* is necessary for infected people.

Currently, the main eradication treatment is quadruple therapy [3, 4]. For *H. pylori*-infected patients with penicillin allergy, proton pump inhibitors, bismuth agents combined with tetracycline, and metronidazole are recommended by international consensus [5, 6]. However, tetracycline has been gradually withdrawn from clinical application. Second-generation tetracyclines have been widely used in clinical practice instead of tetracycline. Besides, in *“Sixth National Guidelines for the Treatment of Helicobacter pylori Infection 2022”*, experts propose that semi-synthetic tetracycline can be used in eradication treatment instead of tetracycline. Minocycline, a second-generation semi-synthetic tetracycline, has better pharmacokinetic characteristics than the first-generation tetracycline. In-vitro sensitivity studies have also shown that the sensitivity of *H. pylori* to minocycline is similar to that of tetracycline [7]. In addition, minocycline has also been proven to be effective and safe in the latest anti-*H. pylori* clinical trials [8]. Consequently, the new quadruple anti-*H. pylori* regimen consisting of minocycline combined with metronidazole, bismuth agents, and proton pump inhibitors, is an excellent alternative for patients with amoxicillin allergy or those who are unable to use amoxicillin for various reasons.

In recent years, many clinical studies have found that minocycline and metronidazole can cause severe side effects. Many patients experience intermittent vomiting and dizziness after starting the new quadruple anti-*H. pylori* regimen consisting of minocycline combined with metronidazole, bismuth agents, and proton pump inhibitors [9]. This may be due to the de-inhibition of the central vestibular regulatory mechanism caused by minocycline [10]. In addition to this, metronidazole is a nitroimidazole derivative that easily penetrates the blood-brain barrier, causing dizziness, headache, ataxia, and other neurological symptoms. Therefore, for clinicians, effective methods to reduce the incidence of adverse reactions to the minocycline regimen are urgently required.

Vitamin B6 is an essential water-soluble vitamin for humans, which can affect the metabolism of many substances. It is often used to prevent various neuropathies, relieve vomiting, and relieve symptoms such as hand and foot neuritis. Vitamin B6 is currently recommended for the treatment of the side effects of oral contraceptives and to relieve symptoms such as nausea, headache, vomiting, dizziness, depression, and irritability [11]. In a double-blind trial, Claussen et al. found that when vitamin B6 was used in combination with minocycline rather than minocycline alone, symptoms of vertigo and nausea, nystagmus, and vestibular spinal cord disorder during stepping and standing were significantly reduced [12]. Besides, previous studies have shown that supplementation of vitamin B6 20 mg ~ 50 mg per day can improve mild cognitive impairment [13, 14]. We speculated that the dose of 20 mg ~ 50 mg per day may be able to stabilize the neurotransmitters in the brain.

These studies have confirmed, to a certain extent, that the application of vitamin B6 can alleviate some neurological symptoms, but there is no study on whether it can alleviate the adverse neurological effects caused by the new anti-*H. pylori* treatment regimen containing minocycline and metronidazole. Based on the above-mentioned research background, our team designed a randomized controlled clinical study to observe whether vitamin B6 can alleviate the adverse reactions caused by the quadruple anti-*H. pylori* treatment regimen containing minocycline and metronidazole.

## Materials and methods

### Study design

This study reports on a randomized controlled trial designed to assess the ability of vitamin B6 to reduce the adverse reactions of the quadruple anti-*H. pylori* regimen containing minocycline and metronidazole. All the patients included in the trial signed an informed consent form. The protocol for this study was approved by the Medical Ethics Committee of Shanghai General Hospital according to the Declaration of Helsinki. The study was registered with Chinese Clinical Trial Registry under the number ChiCTR2100053833 (Study title: Randomized clinical trial: Vitamin B6 reduces the adverse reactions of the quadruple anti-*H. pylori* regimen containing minocycline and metronidazole).

### Study population

Patients who entered the study met all of the following criteria: men or women aged 18–75, who were patients diagnosed with *H. pylori* infection. They also had a positive result of urease test, or histological examination, or ^13^ C-urea breath test during gastroscopy, and they had never received anti-*H. pylori* treatment.

Patients with any one of the following conditions were excluded: those who had used antibiotics, bismuth, or PPI during the past 2 weeks; those who had used non-steroidal anti-inflammatory drugs (NSAIDs) during the last 4 weeks; those who had used drugs that can cause vitamin B6 deficiency, such as isoniazid and antiepileptic drugs, during the past 4 weeks; those who had a history of allergic reactions to metronidazole or tetracycline; those who had clinically significant hepatic, renal, cardiovascular, respiratory, endocrine, or central nervous system disorders; those who were pregnant or nursing mothers; and those who had a history of gastrointestinal surgery.

Furthermore, if the patients had any one of the following conditions, the trial would have been terminated immediately: severe adverse reactions that the patients could not tolerate during the treatment, other diseases that interfered with the outcome of the treatment, being unable to have follow-up sessions, and pregnancy during treatment.

### Study protocol

#### Grouping, treatment, and follow-up

The Jiading Branch of Shanghai General Hospital (Jiading District, Shanghai) carried out the centralized and randomized allocation of patients to either the conventional treatment group or the vitamin B6 supplement treatment group, in a 1:1 ratio. All randomization information was stored securely and only authorized personnel could access it. The data was managed by specialized statisticians. The patients in the conventional treatment group were instructed to take oral rabeprazole enteric-coated tablets 10 mg twice a day, metronidazole tablets 400 mg three times a day, minocycline capsules 100 mg twice a day, and bismuth potassium citrate capsules 220 mg twice a day. The study patients in the vitamin B6 supplement treatment group received oral doses of vitamin B6 20 mg twice a day in addition to the drugs used in the conventional treatment group. The treatment of the two groups was ended after 2 weeks. And the study patients were asked to return at the end of eradication therapy to take a ^13^ C-urea breath test to assess post-treatment *H. pylori* status.

At the beginning of the screening period, the patient demographics were recorded. Vital signs, physical examinations, and clinical laboratory tests were performed. Adverse events, concomitant medications, and treatment compliance were also assessed.

#### Outcome parameters used to evaluate efficacy

Significantly effective: the ^13^ C-Urea breath test was negative and symptoms such as abdominal pain, bloating, acid reflux, and heartburn disappeared completely. Effective: the ^13^ C-Urea breath test was negative, but the patient’s discomfort was not completely relieved. Ineffective: the ^13^ C-Urea breath test was positive and the symptoms did not improve or became worse. Eradication rate = number of people who had significantly effective + effective results / total number of people × 100%.

#### Adverse reaction assessment

The primary adverse reactions of the quadruple anti-*H. pylori* drugs include symptoms of the central nervous system such as dizziness and drowsiness and gastrointestinal symptoms such as nausea, vomiting, abdominal pain, and bloating. We mainly evaluated the overall incidence of adverse reactions (including the application of VSS-C scale scores to assess 14-day central nervous system symptoms and open-ended questions to evaluate gastrointestinal symptoms).

After the overall assessment, we classified the adverse reactions as mild (did not interfere with daily activities), moderate (affected daily activities), and severe (severely affected daily activities and withdrawal).

#### Sample size

According to the previous data with a small sample size, the estimated rate of adverse events around 70% in the conventional treatment group and 55% in the vitamin B6 supplement treatment group, it was calculated [15] that 143 cases were needed in the conventional treatment group, and according to the 1:1 allocation, a total of 286 cases were needed in the two groups. Ultimately, we decided to include 300 patients.

### Statistical analysis

The team used SPSS (version 26; Windows), along with a statistical analysis plan, to calculate the mean and standard deviation of quantitative data and the percentage of qualitative data.

The chi-square test was conducted based on the total eradication rate of *H. pylori* infection. The incidence of adverse reactions, severity of adverse reactions, and curative effects were compared between the different treatment groups. A value of P < 0.05 indicated that the difference was statistically significant.

## Results

### Patient population

**Fig. 1:**
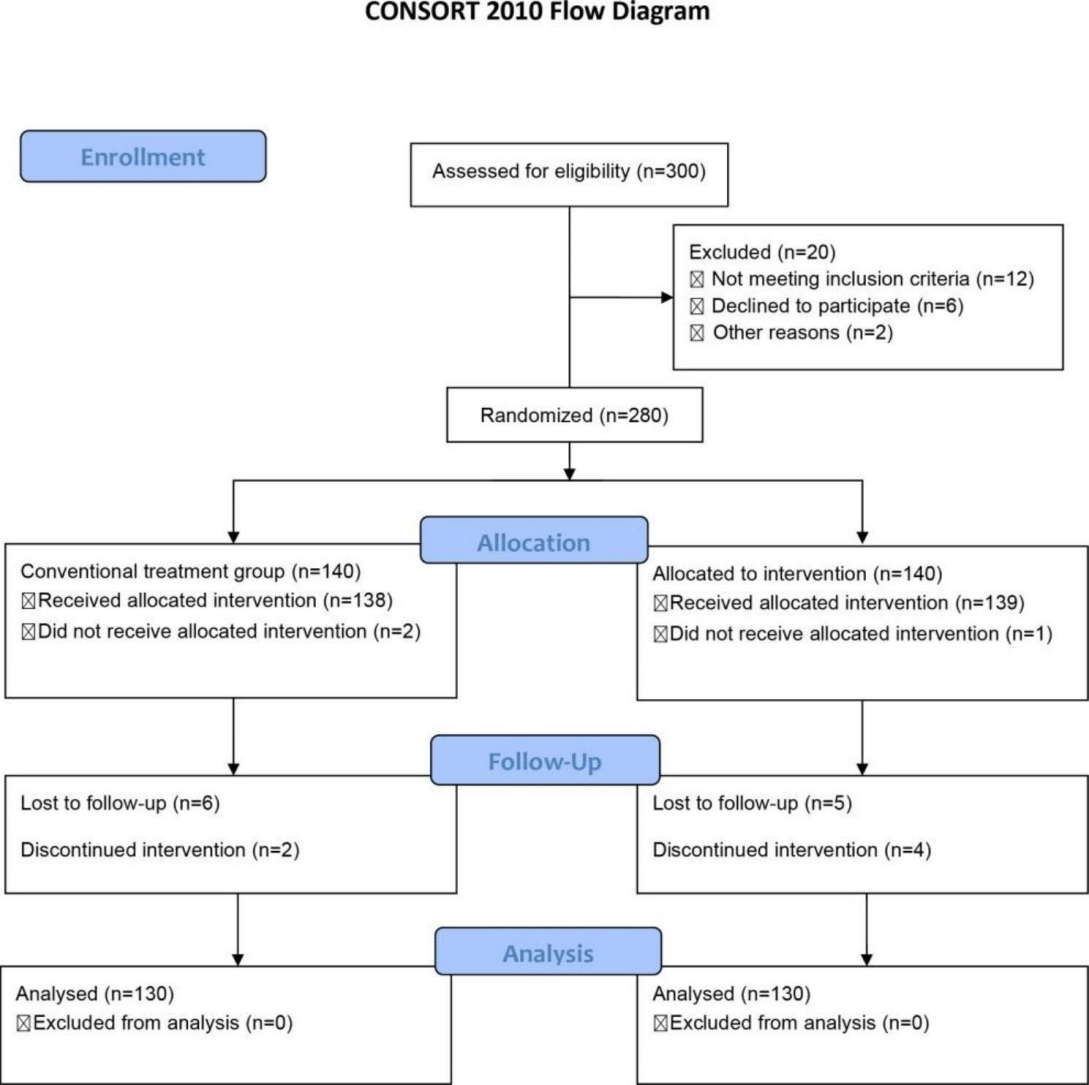
The design of the treatment groups

We recruited 300 patients who were diagnosed with *H. pylori* infection at Jiading branch of Shanghai General Hospital from August 2020 to June 2021. Among the 300 patients who were screened, 20 patients were unqualified according to the inclusion and exclusion criteria. The remaining 280 patients were randomly placed, in a 1:1 ratio, into either the conventional treatment or the vitamin B6 supplement treatment groups. During the process, 10 patients in each group withdrew from the trial. Finally, we analyzed the data of these 260 patients who completed the trial. Figure 1 summarizes the patient characteristics, and Table 1 summarizes the demographic and baseline characteristics of the patients. As shown in Table 1, no significant differences were observed in the demographic characteristics between the different treatment groups.

**Table 1:** Baseline condition of subjects

Variable	Conventional treatment group (N = 130)	Vitamin B6 supplement treatment group (N = 130)	Statistics	*P* value
Age (mean ± SD)	44.13 ± 13.82	45.47 ± 13.52	0.789	0.431
Gender			1.867	0.172
Female	74(56.92%)	63(48.46%)		
Male	56(43.08%)	67(51.54%)		
BMI (mean ± SD)	23.18 ± 2.26	22.79 ± 2.28	-1.367	0.173
Creatinine (mean ± SD)	62.65 ± 9.26	60.70 ± 9.11	-1.635	0.103
History of smoking and/ or alcohol; yes	35(26.92%)	38(29.23%)	0.171	0.679
History of drug allergy; yes	8(6.15%)	9(6.92%)	0.063	0.802

### Efficacy analysis

The percentages of eradiation rate over the 2-week treatment period were 61.54% in the conventional treatment group and 56.15% in the vitamin B6 supplement treatment group. There was no statistical difference between the two groups (X^2^ = 0.778, *P* = 0.378 > 0.05), which confirmed that the vitamin B6 supplement treatment regime was not inferior to the conventional treatment regime (Table 2). Vitamin B6 does not affect the efficacy of conventional regimen.

**Table 2:** Comparison of efficacy between different treatment groups

Variable	Conventional treatment group (N = 130) N(%)	Vitamin B6 supplement treatment group (N = 130) N(%)	Statistics	*P* value
Ineffective rate	50(38.46%)	57(43.85%)	0.778	0.378
Effective rate	71(54.62%)	62(47.69%)	1.247	0.264
Significantly effective rate	9(6.92%)	11(8.46%)	0.217	0.642
Eradication rate	80(61.54%)	73(56.15%)	0.778	0.378

### Adverse reaction analysis

Adverse reaction analysis was performed in the 260 patients included in the clinical trial. In the conventional treatment group, the incidence of adverse reactions was 74.62%, which was significantly higher than the 56.92% observed in the vitamin B6 supplement treatment group. The significant difference between the two groups was confirmed (X^2^ = 9.037, *P* = 0.003 < 0.05). To quantitatively evaluate the overall adverse reactions, we used the VSS-C scale to assess the central nervous system symptoms over 14 days and used open-ended questions to evaluate gastrointestinal symptoms in all the patients enrolled. As shown in Table 3, in the vitamin B6 supplement treatment group, 6.15% of patients had moderate adverse reactions, and 4.62% of patients had severe adverse reactions. These values were significantly lower than those in the conventional treatment group (P < 0.05).

The most common adverse reactions in both treatment groups, as classified by system organ class, were central nervous system symptoms. As shown in Table 4, the incidence of moderate or severe cases of neurological symptoms in vitamin B6 supplement group was 14.63%, which was lower than 58.7% in conventional treatment group (P< 0.05). The incidence of moderate or severe cases of gastrointestinal symptoms in the vitamin B6 supplement group was 0%, which was decreased from 33.33% in conventional treatment group (P< 0.05). Besides, there were many patients with central nervous system symptoms and gastrointestinal symptoms at the same time. The ratio was declined in vitamin B6 supplement treatment group compared with conventional treatment group (13.83% vs. 34.62%, P < 0.01).

**Table 3:** Comparison of adverse reactions between different treatment groups

Variable	Conventional treatment group (N = 130) N(%)	Vitamin B6 supplement treatment group (N = 130) N(%)	Statistics	*P* value
Asymptomatic	33(25.38%)	56(43.08%)	9.037	0.003
Adverse reactions; yes	97(74.62%)	74(56.92%)	9.037	0.003
Mild	39(30.00%)	60(46.15%)	7.194	0.007
Moderate	33(25.38%)	8(6.15%)	18.098	< 0.001
Severe	25(19.24%)	6(4.62%)	13.222	< 0.001

**Table 4:** Adverse reactions reported in the different treatment groups

Variable	Conventional treatment group (N = 130)	Vitamin B6 supplement treatment group (N = 130)	Statistics	*P* value
Central nervous system symptoms	46/130(35.38%)	41/130(31.54%)	0.432	0.511
Mild	19/46(41.30%)	35/41(85.37%)	17.876	< 0.001
Moderate	15/46(32.61%)	4/41(9.76%)	6.632	0.010
Severe	12/46(26.09%)	2/41(4.87%)	7.222	0.007
Gastrointestinal symptoms	6/130(4.62%)	15/130(11.54%)	4.200	0.041
Mild	4/6(66.67%)	15/15(100%)	5.526	0.019
Moderate	2/6(33.33%)	0/15(0.00%)	5.526	0.019
Severe	0/15(0.00%)	0/15(0.00%)	-	-
Both central nervous system symptoms and gastrointestinal symptoms	45/130(34.62%)	18/130(13.85%)	15.27	< 0.001
Mild	16/45(35.56%)	10/18(55.56%)	2.122	0.145
Moderate	16/45(35.56%)	4/18(22.22%)	1.055	0.304
Severe	13/45(28.88%)	4/45(22.22%)	0.29	0.590

## Discussion

The present study was designed to evaluate whether vitamin B6 can alleviate the adverse reactions caused by the quadruple anti-*H. pylori* treatment regimen containing minocycline and metronidazole. In the study by Villegas Salas et al., the non-toxicity of vitamin B6 was confirmed after exploring whether vitamin B6 can reduce the adverse effects of oral contraceptives [11]. There are no reported cases of vitamin B6 deficiency during tetracycline or metronidazole treatment. In our study, there was no statistical difference in the eradication rate of *H. pylori* infection between the conventional treatment group and the vitamin B6 treatment group, which also confirmed the non-toxicity of vitamin B6 during eradication treatment.

Minocycline combined with metronidazole, bismuth, and PPI is an excellent treatment for patients with *H. pylori* infection who are allergic to penicillin. However, patients who use minocycline and metronidazole are more likely to have symptoms such as nausea, epigastric discomfort, and dizziness. As shown in Table 4, the adverse reaction rate of the conventional regimen containing minocycline and metronidazole was 74.62%. Although, through overall assessment, most patients had only mild to moderate adverse reactions, 19.24% of the patients receiving this treatment had severe adverse reactions. This may be due to the brainstem vertigo induced by minocycline, which is caused by the de-inhibition of the central vestibule due to the instability of the supervised gamma-aminobutyric acid (GABA) ring in the medulla oblongata regulation pathway of the pontine [12]. In the vitamin B6-supplemented treatment group, the incidence of adverse reactions dropped to 56.92%. In addition, among these patients, only 6.15% of the patients had moderate adverse reactions, and 4.92% of the patients had severe adverse reactions, which was significantly lower than those in the conventional regimen group. Besides, we counted the main adverse reactions in different system organ class in Table 4. It can be seen that the severity of central nervous system symptoms or the gastrointestinal symptoms was significantly decreased in the vitamin B6 group, which was consistent with Table 3. After vitamin B6 supplementation, the proportion of moderate to severe central nervous system symptoms decreased from 58.7 to 14.63%. And, the proportion of moderate to severe gastrointestinal reactions decreased from 33.33 to 0%.

According to previous research, we speculate that the alleviation of adverse reactions may be because vitamin B6 drives the production of GABA in the brain by regulating glutamate decarboxylase [16] and reversing the de-inhibition of the central vestibule, thereby alleviating the adverse reactions caused by minocycline. Pyridoxal phosphate (PLP) is the active form of vitamin B6 in the human body. It undergoes a condensation reaction with the amino group of amino acids which forms the basis of most of the enzyme catalyzed reactions for which PLP is required. Glutamate decarboxylase and GABA-transaminase are only active under the action of pyridoxal phosphate. The neurotransmitter GABA in brain is formed from glutamate through the action of glutamate decarboxylase. And GABA is metabolized by the action of GABA-transaminase. Besides, pyridoxal phosphate is also involved in the activation of branched-chain amino acid aminotransferase, which involves in the production of glutamate in the brain [17, 18]. Therefore, vitamin B6 plays a pivotal role in the metabolic cycle of GABA and glutamate, which can maintain the neural homeostasis in the brain. When the minocycline regimen affects the GABA ring in the brain, vitamin B6 supplementation may be stabilize the ring to a certain degree. The specific biochemical and pharmacological mechanisms involved need to be further explored.

Our study has several limitations. First, it was a single-center clinical study with a small sample size. Although based on our research data, the sample size has met to the minimum standard. We should appropriately expand the sample size while considering the missing rate. And further double-blind trial studies with larger sample sizes are needed in the future. Second, we speculate that the mechanism of vitamin B6 of reducing adverse reaction may be related to the production of GABA in the brain, which needs to be proved by further experiments. Third, previous studies suggest that the eradication rate of minocycline regimens is up to 80% [19]. However, the eradication rate in our research was 60%. The reason for the difference may be the resistance rate of tetracycline or metronidazole being high in the area where the trial was conducted. Nevertheless, this is our speculation. The clear causes may need further investigation to clarify. Forth, in this study we only explored the effect of a dose of vitamin B6 20 mg twice a day, we should try to supply different doses of vitamin B6 to explore the optimal dose to reduce adverse reactions of conventional treatment.

To the best of our knowledge, this is the first study to explore whether vitamin B6 can alleviate the adverse reactions of the quadruple anti-*H. pylori* regimen containing minocycline and metronidazole. The significance is that vitamin B6 can be added in minocycline regimen for the *H. pylori*-infected individuals, who has to choose this regimen for various reasons, to alleviate dizziness, nausea and other related symptoms.

In conclusion, we have shown that the effect of *H. pylori* eradication did not differ between the conventional treatment group and vitamin B6 supplement treatment group. Nevertheless, in the vitamin B6 supplement treatment group, the incidence of adverse reactions was significantly lower than that in the conventional treatment group.

### Electronic supplementary material

Below is the link to the electronic supplementary material.

Supplementary Material 1

Supplementary Material 2

## Data Availability

All data analyzed during this study were included in supplementary information files.

## Declarations

### Trial registration

The study was retrospectively registered with Chinese Clinical TrialRegistry (http://www.chictr.org.cn/usercenter.aspx) under the number ChiCTR2100053833 in November 2021. (Study title: Randomized clinical trial: Vitamin B6 reduces the adverse reactions of the quadruple anti-*H. pylori* regimen containing minocycline and metronidazole)
